# Expression of Foot-and-Mouth Disease Virus Capsid Proteins in Silkworm-Baculovirus Expression System and Its Utilization as a Subunit Vaccine

**DOI:** 10.1371/journal.pone.0002273

**Published:** 2008-05-28

**Authors:** Zhiyong Li, Yongzhu Yi, Xiangping Yin, Zhifang Zhang, Jixing Liu

**Affiliations:** 1 Key Laboratory of Animal Virology of Ministry of Agriculture, State Key Laboratory of Veterinary Etiological Biology, Lanzhou Veterinary Research Institute, Chinese Academy of Agriculture Sciences, Lanzhou, Gansu, China; 2 Biotechnology Research Institute, Chinese Academy of Agricultural Sciences, Beijing, China; Federal University of Sao Paulo, Brazil

## Abstract

**Background:**

Foot-and-mouth disease (FMD) is a highly contagious disease of livestock that causes severe economic loss in susceptible cloven-hoofed animals. Although the traditional inactivated vaccine has been proved effective, it may lead to a new outbreak of FMD because of either incomplete inactivation of FMDV or the escape of live virus from vaccine production workshop. Thus, it is urgent to develop a novel FMDV vaccine that is safer, more effective and more economical than traditional vaccines.

**Methodology and Principal Findings:**

A recombinant silkworm baculovirus Bm-P12A3C which contained the intact P1-2A and 3C protease coding regions of FMDV Asia 1/HNK/CHA/05 was developed. Indirect immunofluorescence test and sandwich-ELISA were used to verify that Bm-P12A3C could express the target cassette. Expression products from silkworm were diluted to 30 folds and used as antigen to immunize cattle. Specific antibody was induced in all vaccinated animals. After challenge with virulent homologous virus, four of the five animals were completely protected, and clinical symptoms were alleviated and delayed in the remaining one. Furthermore, a PD_50_ (50% bovine protective dose) test was performed to assess the bovine potency of the subunit vaccine. The result showed the subunit vaccine could achieve 6.34 PD_50_ per dose.

**Conclusion:**

The results suggest that this strategy might be used to develop the new subunit FMDV vaccine.

## Introduction

Foot-and-mouth disease (FMD) is an economically important disease of domestic and wild cloven-hoof animals including cattle, swine, goat, sheep and buffalo. It can result in great reduction of productivity in adult animals and death in young animals. FMD is endemic in parts of Asia, Africa, the Middle East and South America (with sporadic outbreaks in other “free areas”). In countries affected by the disease, livestock trade and animal products have been impacted. Even in developed countries and areas, outbreak of FMD would greatly affect the economy. In 2001, the outbreak of FMD in England brought a loss of 8 billion dollars [1], and the consequent occurrence in Holland killed 0.2 million animals [2]. In addition, the 1997 outbreak in Taiwan brought a loss of about 3.6 billion dollars to its exports [3]. Therefore, controlling bath endemic and epidemic FMD has become a global concern in livestock raising.

At present, vaccination is a major means of FMD control in most endemic areas. The present method of FMDV vaccine production is this: The virus is propagated in BHK-21 cell line, concentrated, and chemically inactivated. Although the inactivated vaccine has been shown to be effective, it may lead to new outbreaks of FMD because of either the incomplete inactivation of FMDV in large-scale production or the escape of the live virus from vaccine production workshops [4]. Therefore several expression systems such as *E.coli* [5], transgenic plant [6], yeast [7], adenovirus vector [8]–[12], vaccinia virus vector [13], and DNA vaccine [14], have been used for expression of FMDV antigen to prepare subunit vaccines. But such methods have problems such as poor immunogenic capability or low efficiency. Adenovirus based vaccine known for its best protective effects can protect 5 of 5 vaccinated cattle, but this vaccine is still unacceptable because of safety problem and preservation difficulty. The baculovirus expression system, a valuable expression system to produce virus-like particles, has successfully produced many kinds of empty viral capsids [15], [16], such as rabbit hemorrhagic disease virus, Norwalk-like viruses,SARS and so on [17], [18], [19]. Compared to the baculovirus expression system (AcNPV-*Sf* cell), silkworm-baculovirus expression system has distinct advantages [20], [21]. First, expression levels in silkworm are 50–1000 times higher than that in insect cell lines. Second, silkworm don not have any pathogens that can cross infect with vertebrates and animal serum is not needed to produce foreign proteins in this expression system, so that the expressed antigens are safer to vertebrates. In view of all these advantages, the silkworm-baculovirus expression system was employed for expression of intact P1-2A, 3C coding regions of FMDV Asia I/HNK/CHA/05. All five cattle that were vaccinated with diluted expression antigen were induced specific antibody, four of which were considered completely protected. Furthermore, the PD_50_ (50% bovine protective dose) value of the subunit vaccine was 6.34 in bovine potency test.

## Results

### Construction of the recombinant virus Bm-P12A3C

Intact P1-2A and 3C protease coding regions from FMDV Asia I/HNK/CHA/05 strain were amplified by RT-PCR and inserted into the transfer vector pVL1393 to generate plasmid pVL-P12A3C. The plasmid was digested with *Bam*H I/*Eco*R I to generate fragments of 2.3 kb and 10 kb, while fragments of 0.7 kb and12 kb were generated with *Eco*R I/*Bgl* II. Based on the length of these fragments, it was confirmed that the full target fragment (P12A3C) was correctly incorporated into the transfer vector and that the expression cassette was located downstream of *polyhedrin* promoter. Sequence analysis indicated that the P12A3C was 3,024 bp containing full P1-2A and 3C genes and partial 2B,3B genes.

*Bm*-N cell line was co-transfected with baculoviral transfer plasmid pVL-P12A3C and linearized BmBacPAK-6 DNA. The supernatant was collected 4 days post transfection as the viral stock for screening of recombinant virus. Twenty four isolated viral plaques from the plaque assays were cultivated in a 24-well plate and were inoculated into silkworms. The plaque of recombinant virus expressed at maximal activity was selected to purify. The pure recombinant virus from the last round was used as stock virus and confirmed to contain the full expression cassette. The recombinant virus Bm-P12A3C was used to express FMDV protein in cells or silkworm.

### Expression of polyprotein in *Bm*-N cell

**Figure 1:**
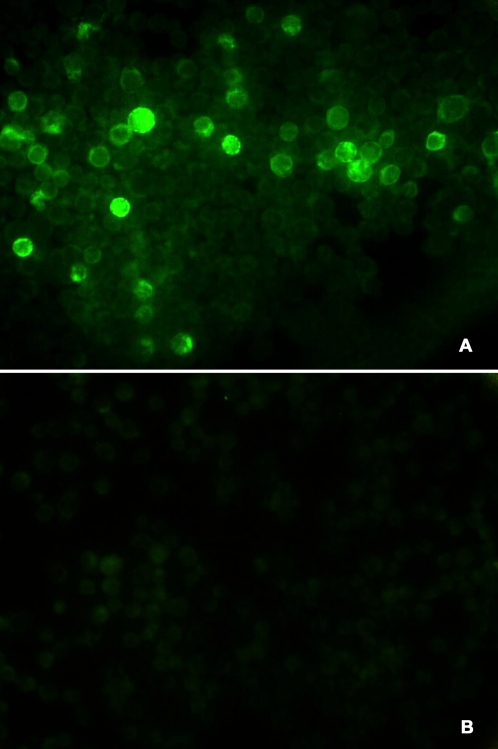
Expression of FMDV polypeptides in *Bm*-N cells was analysed by IFAT. (A) *Bm*-N cells infected with Bm-P12A3C. (B) *Bm*-N cells infected with BmBacPAK-6.

The expression of polyprotein in *Bm*-N cells was analyzed by IFAT and sandwich-ELISA. IFAT pictures demonstrated that *Bm*-N cells infected with Bm-P12A3C produced specific fluorescence, while only very weak background fluorescence appeared in the control cells (Fig. 1). This indicated that polyprotein was indeed expressed in *Bm*-N cell. The sandwich-ELISA results indicated that the FMDV antigen in Bm-P12A3C infected cells was expressed at levels about equivalent to the positive control, but was not detected in BmBacPAK-6 infected cell lysate.

### Expression of polyprotein in silkworm

**Figure 2:**
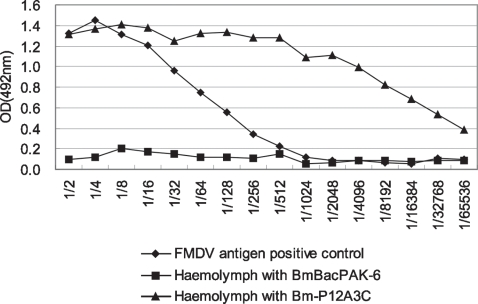
Expression of FMDV polypeptides in silkworm larvae was estimated by the sandwich-ELISA. The haemolymph was diluted with two-fold series.

The Bm-P12A3C virus was inoculated 1,000 silkworms. The dying silkworm's haemolymph was collected (about 4–5 d post infection, corresponding with rearing mean temperature about 25–27°C). Sandwich-ELISA was conducted to evaluate the expressed antigen in silkworm. The results indicated that OD value of the harvested haemolymph from silkworm infected by Bm-P12A3C decreased as the dilution rate increased, which was in good agreement with variation of positive control of FMDV antigen. The expression yield was about 100 fold (positive antigen: 1/32,0.964; expressed antigen in haemolymph: 1/4096, 0.995) more than the positive control (BHK-21 cell vaccine which had a PD_50_ value of 3.6), but was not detectable in the negative control (BmBacPAK-6 infected silkworm's haemolymph) (Fig. 2).

**Figure 3:**
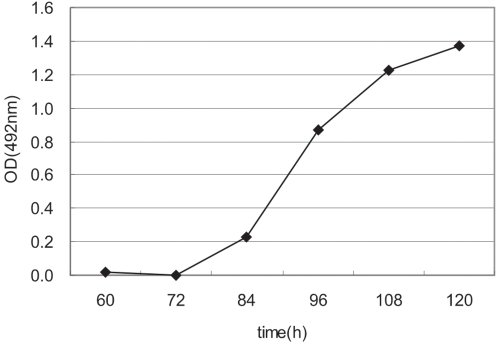
The time courses of FMDV polyprotein expressed in silkworm. The larval haemolymph was collected on ice every 12 h from 60 hpi. The FMDV antigen in haemolymph was analyzed by the sandwich-ELISA. The hemolymph was diluted with 1,000 folds dilution.

In order to determine the time course of expressed antigen in silkworm and the optimum acquisition time for large scale's production, haemolymph from 10 silkworms was harvested every 12 h beginning at 60 hpi and stored at −20°C. Subsequently, the haemolymph was diluted to 1000 folds for detection of expression products (Fig. 3). There was a little at 60 hpi, and the accumulation of recombinant products were dramatically increased from 84 hpi and kept at the high levels during the late phase of infection. So, expressed antigen could be harvested at 108–120 hpi (at the condition about the mean rearing temperature of 25°C).

### The anti-FMDV antibody in cattle

Cattle were vaccinated by the vaccine prepared from 1/30 diluted expressed antigens (the quantity of antigen was about 3 folds of the BHK-21 cell vaccine). LPBE-antibody titer was determined at 7, 14, 21 and 28 dpv following the LPBE method. It was found that all five cattle vaccinated with Bm-P12A3C antigen developed a detectable FMDV-antibody response at 7 dpv, and dramatically reached to high level at 14 dpv. By 21and 28 dpv, the antibody level was maintained at the same level or higher, and reached to a titer of 360 in cattle No33 and No50. In contrast, antibody level in the two control cattle (vaccinated with vaccine prepared from BmBacPAK-6) was not boosted (Table 1).

**Table 1:** FMDV-specific antibody response after vaccination with Bm-P12A3C's.

Animal	Vaccine^a^	LPBE-antibody^b^	dpv^c^			
number		−7	7	14	21	28
33	Bm-P12A3C	<8	32	180	360	360
124	Bm-P12A3C	<8	20	90	90	90
122	Bm-P12A3C	<8	20	45	45	45
45	Bm-P12A3C	<8	20	90	90	90
50	Bm-P12A3C	<8	45	360	360	360
2	BmBacPAK-6	<8	<8	<8	<8	<8
11	BmBacPAK-6	<8	<8	<8	<8	<8

a Bovines were vaccinated with vaccine prepared from 30 folds diluted expressed antigens (Bm-P12A3C) or the control (BmBacPAK-6) and challenged 28 days later.

b FMDV-specific antibody titer reported as the serum dilution by LPBE method.

c Days postchallenge.

Furthermore, sera were analyzed for neutralizing antibodies against FMDV (Table 2). While the two control cattle could not develop any neutralization antibodies against FMDV, all five cattle vaccinated with Bm-P12A3C antigen developed a FMDV-specific neutralizing antibody response in 14pv and maintained at the same level or higher in 28 dpv. The result was in agreement with the LPBE-antibody titer.

**Table 2:** Results of neutralizing antibody response against FMDV after inoculation.

Animal	#Vaccine^a^	neutralizing antibody^b^	dpv^c^	[Table-fn nt106]	[Table-fn nt106]	[Table-fn nt106]
number		−7	7	14	21	28
33	Bm-P12A3C	<8	16	64	90	90
124	Bm-P12A3C	<8	8	64	64	64
122	Bm-P12A3C	<8	8	32	32	32
45	Bm-P12A3C	<8	8	32	32	32
50	Bm-P12A3C	<8	8	64	90	90
2	BmBacPAK-6	<8	<8	<8	<8	<8
11	BmBacPAK-6	<8	<8	<8	<8	<8

a Bovines were vaccinated with vaccine prepared from 30 folds diluted expressed antigens (Bm-P12A3C) or the control (BmBacPAK-6).

b FMDV-specific antibody titer reported as the serum dilution by neutralization tests.

c Days postchallenge.

### Challenge with FMDV Asia I/HNK/CHA/05

All vaccinated cattle were challenged with 10,000 BID_50_ of Asia I/HNK/CHA/05 at 28 dpv. Body temperature, mouth and feet were observed consecutively for ten days to evaluate the incidence of disease (Table 3). Four of the five cattle were considered completely protected. Only one vaccinated cattle, No45, developed lesions. The lesions were detectable by 6 dpc and the clinical signs were less severe compared to control group. By contrast, vesicles developed in the control animals by 2 dpc at the sites of all feet and mouth. This indicated that antigen produced in silkworm could be effectively protective.

**Table 3:** Protection and clinical signs in cattle after challenge with FMDV Asia I/HNK/CHA/05.

Animal number #	vaccine	Days of onset of pyrexia^a^	Duration of Pyrexia(days)	Lesion scores^b^	Protection^c^
33	Bm-P12A3C	-	-	-	+
124	Bm-P12A3C	-	-	-	+
122	Bm-P12A3C	-	-	-	+
45	Bm-P12A3C	Day 6	2	2	-
50	Bm-P12A3C	-	-	-	+
2	BmBacPAK-6	Day 2	3	4+mouth	-
11	BmBacPAK-6	Day 2	3	4+mouth	-

a Pyrexia defined as body temperature 40°C.

b The lesion score is the number of feet on which the cattle exhibited.

c Protection was determined that cattle did not take on the clinical signs of FMD during observation period (10 days post-challenge).

### The result of PD_50_ test

The PD_50_ test was performed to assess the subunit vaccine potency by following the bovine potency test protocol described by the OIE to test the traditional inactivated FMD vaccines. In this research, the result showed the vaccine potency of the batch immunized with the expressed antigens reached 6.34 PD_50_ per dose (Table 4).

**Table 4:** The result of PD_50_ test.

Immunize dose	Rate of protection (%)	PD_50_
1	5/5 (100)	6.34
1/3	4/5 (80)	
1/9	2/5 (40)	

All of cattle were challenged with 10,000 BID_50_ of Asia I/HNK/CHA/05 after vaccination by inoculating the equivalent of a total of 10,000 BID_50_ of Asia I/HNK/CHA/05 intradermally into two sites on the upper surface of the tongue. All cattle were observed for 10 days. Vaccinated animals are protected if they do not develop lesions on the feet and areas other than the injection sites on the tongue. Rate of protection (%) = number of cattle no lesions/total number of cattle. The PD_50_ value was calculated by the Reed-Muench method.

## Discussion

FMD still threatens world livestock production. Seven distinct serotypes of FMDV have been identified, named A, O, C, Asia I, SAT I, SAT and SAT with none cross immune protection occurring between them. The FMDV serotype Asia which was first isolated in Pakistan is epidemic within Southeast Asia and Indian peninsula, disseminating among Near East, Middle East and Far East [22]. In March 2005, FMDV serotype Asia was found in HongKong (Asia I/HNK/CHA/05 strain). Subsequently, this type of the virus was reported from mainland of China in April 2005[23]. The P1 sequence of Asia 1/HNK/CHA/05 isolate was aligned and compared with 9 reference sequences. The result confirmed that Asia 1/HNK/CHA/05 has a high identity with nine Asia I reference sequences from 85.9 to 92.6% [24].

Expression products of baculovirus expressing system are generally considered to be well immunogenic and possess the ability to assemble empty viral capsid. When the same FMDV expression cassette were expressed in *E.coli* and baculovirus expression system, the expression products from baculovirus excels that from *E.coli* in terms of the immunogenic [25] and protective effects [26]. Empty capsid comes into being only when capsid precursor P1-2A, protease L and 3C coding region from FMDV O1K serotype were all expressed in baculovirus expressing system(AcMNPV-*Sf* cell). Truncated protease L can not be self-cleaved from VP0. But, the expressed protease L is harmful to host cell growth, reducing the expression efficiency [27]. In adenovirus expression system, P12A3C expression cassette, including full structure of P1-2A,3C and portion of 2B and 3B, can be expressed and assembled into empty capsids [8]. Myristoylation of the animo terminus of P1-2A is of great importance to the assembly of viral particles [28]. It has been reported that the expression products of AcMNPV-*Sf* cell can be myristoylated well [29]. Based on the above studies, and using the design previously published by Mayr et al [8], the P12A3C expression cassette of FMDV serotype Asia I was constructed. After two sorting rounds of recombinant virus and measurements of expression efficiency for more than 20 viral clones, the over-expressed recombinant virus Bm-P12A3C was obtained. It can express with very high efficiency in the hyperexpression variety of silkworm (JY1). The specific antigen produced per milliliter in silkworm haemolymph at least 100 folds more than the BHK-21 cell vaccine which had a PD_50_ value of 3.6.

Because cattle are the most important economic and susceptible cloven-hoof animal, we designed an experiment to verify whether this produced antigen can be used for preparing a cattle FMD vaccine. We followed the bovine potency test protocol described by the OIE to test this subunit vaccine potency. We used 1/30 diluted dosage to vaccinate five cattle and two controls were vaccinated with vaccine prepared from BmBacPAK-6's. By two weeks post vaccination, the antibody level of the five vaccinated cattle reached a high titer. The antibody level has some ascension but maintained thereafter two weeks, while the control group maintained lower than titer 8. After virulent homologous virus challenge, four of the five were considered protected, and one delayed the disease and ease the clinical symptom, but two unvaccinated cattle developed lesions on all the feet and in the inside of mouth on the second day. Cell-mediated -immune response was probably involved in the protection: that would explain why animal 45 has the same neutralizing antibody titers as 122 but is not protected. This demonstrated that the expression products from silkworm- baculovirus expression system were immunogenic as well. Based on above result, we did the PD50 test to assess the bovine potency of the subunit vaccine. When employed for routine prophylactic use, the vaccine should contain at least 3 PD_50_ per dose for cattle by OIE recommended. The result showed the subunit vaccine potency could get 6.34 PD_50_ a dose for cattle. This leads to a conclusion that it is feasible to use the silkworm-baculovirus expression system for FMD vaccine production.

## Materials and Methods

### Viruses and cell lines

FMDV Asia I/HNK/CHA/05 strain (GenBank accession number EF149010) was propagated in BHK-21 cell line, isolated, and preserved in Lanzhou Veterinary Research Institute of Chinese Academy of Agriculture Sciences. The parental virus BmBacPAK-6 (Chinese patent: 1242428), *Bm*-N cell line and silkworm variety JY1 used for the experiment were maintained in Biotechnology Research Institute of Chinese Academy of Agriculture Sciences. The BmBacPAK-6 and recombinant virus were maintained in *Bm*-N cells at 27°C in TC-100 insect medium (Sigma) supplemented with 10% heat-inactivated fetal bovine serum (Invitrogen).

### Construction and screening of recombinant baculovirus

Genomic RNA was extracted from the viral supernatant with RNeasy (Qiagen) and used immediately for cDNA synthesis. cDNA synthesis was performed with Superscript II reverse transcriptase (Invitrogen). PCR was used to amplify the Intact P1-2A and 3C protease coding regions from the cDNA using two pairs of specific primer:

Forward primer for P1-2A: 5′-ATAGGATCCACCATGGGAGCCGGGCAATCCAGCC-3′ (*Bam*H I site was introduced)

Reverse primer for P1-2A 5′-CGCGAATTCTGACATGTCCTCCTGCATCTGGTTG-3′ (*Eco*R I site was introduced)

Forward primer for 3C:5′-GCGGAATTCAAGAAACCTGTCGCTTTGAAAGT-3′ (*Eco*R I site was introduced)

Reverse primer for 3C:5′ATAAGATCTCTACTCGTGGTGTGGTTCGGGAT-3′ (*Bgl* II site was introduced)

The PCR products were separated by 1% Agarose gel electrophoresis. The target fragments of P1-2A and 3C were inserted into baculoviral transfer vector pVL1393 using *Bam*H I/*Eco*R I and *Eco*R I/*Bgl* II sites following the routine protocols to generate a transfer plasmid. The whole P1-2A and 3C coding regions in transfer plasmid was sequenced and named pVL-P12A3C.

The baculoviral transfer plasmid pVL-P12A3C was co-transfected with linearized Bm-BacPAK6 DNA into *Bm*-N cells by liposome-mediated method using transfection reagent lipofectamin 2000 (Invitrogen) [30]. The co-transfection supernatant was subject to plaque assays to screen the individual viral plaques. PCR amplification was conducted to confirm that the P1-2A and 3C genes had been incorporated into the baculoviral genome. Primers were designed as follows: Sense: 5′-ACTGTTTTCGTAACAGTTTTGTAA-3′ and Reverse: 5′-CTACTCGTGGTGTGGTTCGGGAT-3′. Another two rounds of screen were performed and the pure recombinant virus was used to generate high titer viral stocks for expression.

### Expression of FMDV polyprotein in *Bm*-N cells

The expression of FMDV polyprotein in *Bm*-N cells infected with Bm-P12A3C was analyzed by immunofluorescence test (IFAT) and sandwich-ELISA. The Bm-P12A3C was multiplied in *Bm*-N cells. *Bm*-N cells (2.0×10^5^) were cultured on cover slips and inoculated at a MOI of 10 pfu with Bm-P12A3C. After 48 hours post infection (hpi), IFAT was conducted to analyze the expression of FMDV proteins. Cells were then rinsed with PBS for 1 or 2 times and fixed in 100% cold acetone (−20°C for 30 min). Samples were incubated with rabbit serum against FMDV (37°C for 30 min) in humid box, washed with PBS for five times, and then stained with fluorescein-conjugated goat anti-rabbit serum at 37°C for 30 min. The cover slips were coated with glycerin and observed on an Olympus fluorescence microscope. *Bm*-N cells infected with BmBacPAK-6 were used as control.

When *Bm*-N cells infected with Bm-P12A3C were partial floating, they were detached and collected (about 72 hpi), the cells pellet was freezed and thawed at -70/37°C in PBS for three times and centrifuged at 10,000g for 5 min at 4°C. The supernatant was tested using the sandwich-ELISA method. 96-well plat-bottomed plates (Costar) were coated with the rabbit serum against FMDV overnight at 4°C and blocked with defatted milk powder for 1h. Then the plates were washed five times. FMDV antigen (from the vaccine which had a PD_50_ value of 3.6), lysates of *Bm*-N cells with Bm-P12A3C and BmBacPAK-6 infected were diluted in a two-fold series and incubated at 37°C for 1h. Subsequently, the plates were washed thoroughly and guinea pig sera against FMDV was added to each well. The plates were incubated at 37°C for 1 h, and then rabbit anti-guinea pig IgG peroxidase conjugate (Sigma) at 1∶10000 dilution was added and reacted at 37°C for 1 h. Substrate (0.05% H_2_O_2_ plus orthophenylene diamine) was added, reacted for 15 minutes and stopped by the addition of 1M sulphuric acid. Absorbance was determined at 492 nm.

### Expression of polyprotein in silkworm

Early fifth-instar silkworms were infected with the recombinant virus at about 10^5^ pfu per larva. The dying silkworm's haemolymph was collected on ice and stored at −20°C for sandwich-ELISA.

The infected silkworm's haemolymph was collected every 12 h starting at 60 hpi for determining the expression course of target antigen.

### Detection of specific antibody by LPBE and serum neutralization test (SNT)

Silkworm haemolymph was lysed ultrasonically and cell debris was removed by centrifugation. The diluted supernatant was used to produce vaccine. Liquid-phase blocking ELISA(LPBE)(http://www.oie.int/eng/normes/MMANUAL/A_00024.htm) was performed to determine the antibody titer for screening of candidate cattle according to the standard method of World Organization for Animal Health, Office International desEpizooties (OIE) before vaccination. Candidates with a potency lower than 8 were housed in disease-secure isolation facilities in Lanzhou Veterinary Research Institute. Detection kit was prepared by Lanzhou Veterinary Research Institute. Seven cattle (6–8 months old) were immunized by intramuscular inoculation at the site in the neck. Five cattle were vaccinated with 3ml/animal of vaccine with Bm-P12A3C's, while two control cattle were vaccinated with the same dose of vaccine with BmBacPAK-6's.

Cattle serum were collected at 7, 14, 21and 28 days postvaccination (dpv). Antibody against FMDV was detected by LPBE method (as above) and SNT.

For SNT, the sera was diluted in DMEM as two-fold in a 96-well flat-bottomed tissue culture plates (Castor, USA). Virus suspension with a titer of 100 TCID_50_ in 50 ul was added to each sera well and the mixture was incubated for 1 h at 37°C and 5% CO2. 50 ul of BHK-21 cell suspension (1.5×10^6^ ml^−1^) was added to each well and incubated for 4–5 days. Appropriate serum, virus and cell control were included in this test. The plates were observed via microscope for cytopathic effect.

### Challenge with virulent homologous FMDV

According to the descriptions by standard protocol of OIE (http://www.oie.int/eng/normes/MMANUAL/A_00024.htm), all animals were challenged by intradermal inoculation at two sites in the tongue with 10,000 bovine infectious doses (BID_50_) of Asia I/HNK/CHA/05 at 28 dpv. The body temperature of the animals was monitored daily. The restrained animals were carefully examined in the mouth, and feet every day for the first 10 days after challenge.

### PD50 test

We followed the bovine potency test protocol described by the OIE to test this subunit vaccine potency. Three groups of five cattle per group and a control group of two non-vaccinated animals were vaccinated. The vaccinated groups were administered different doses (1 dose, 1/3 dose, 1/9 dose)of the subunit vaccine prepared from diluted expression products and all animals were challenged 3 weeks after vaccination with 10,000 BID_50_ of Asia I/HNK/CHA/05 to the vaccine under test by intradermal inoculation into two sites on the upper surface of the tongue. The animals were observed daily for 10 days after challenge for clinical signs of FMD. Control animals developed lesions on at least three feet. Unprotected animals showed lesions at sites other than the tongue. From each animal protected in each group, the PD_50_ (50% bovine protective dose) content of the vaccine was calculated based on the Reed-Muench method.
